# Neonatal Circumcision Simulation: A Resource for Beginners

**DOI:** 10.15766/mep_2374-8265.11531

**Published:** 2025-06-03

**Authors:** Rakhi Gupta Basuray, Sofia Davila, Jennifer Springer

**Affiliations:** 1 Assistant Professor, Department of Pediatrics, Ohio State University College of Medicine, and Assistant Professor; Physician, Division of Pediatric Hospital Medicine, Nationwide Children's Hospital; 2 Assistant Professor, Department of Pediatrics, College of Medicine, Ohio State University College of Medicine; Physician, Division of Pediatric Hospital Medicine, Nationwide Children's Hospital; 3 Third-Year Resident, Nationwide Children's Hospital

**Keywords:** Circumcision, Simulation, 3D Printing, Deliberate Practice, Neonatal-Perinatal Medicine, Pediatric Urology

## Abstract

**Introduction:**

Neonatal circumcision is one of the most common procedures performed on males in the United States. Though not a required procedure to learn during residency, an interest in learning this skill exists. Simulation provides an opportunity to be exposed to, and gradually master, procedural skills. We report our experience developing a circumcision simulation session, among a large pediatric intern class.

**Methods:**

Participants received an instructional video for asynchronous viewing in advance of an in-person small-group session. Stations for six participants and two facilitators were equipped with Gomco clamp kits and a 15-step checklist. Facilitators demonstrated the neonatal circumcision procedure as participants followed along step-by-step. Bidirectional, real-time feedback was provided. Upon completion, an optional survey allowed participants to anonymously evaluate the course. Descriptive statistics were used for analysis.

**Results:**

Fifty-three interns participated, with a 72% survey completion rate. The simulation session received an overall average rating of 4.9 (based on 5-point Likert scale; 1 = *terrible*, 5 = *excellent*). On several questions (*yes*/*no* response options), respondents unanimously reported that the session allowed identification of areas for targeted improvement, was helpful to experience prior to performing a circumcision on a newborn patient, and improved confidence.

**Discussion:**

The 30-minute neonatal circumcision simulation session was well-received, exposed learners to a desirable procedure, allowed identification of targeted areas for improvement, and increased confidence. Strengths of our session include novel application of a 3D-printed model and a flexible framework that can be adapted to suit a variety of settings, learners, and available resources.

## Educational Objectives

By the end of this activity, learners will be able to:

1. Demonstrate steps of neonatal circumcision via Gomco clamp.
2. Self-identify steps of neonatal circumcision more technically challenging to perform, to use toward deliberate practice.
3. Increase confidence in performing neonatal circumcision.

## Introduction

Neonatal circumcisions are performed on approximately 80% of male infants in the United States, with wide variability between ethnic populations.^1^ Though the American Academy of Pediatrics has not recommended that circumcision be performed routinely, parents are supported in their choice to pursue neonatal circumcision for male newborns.^2^ The ACGME does not require any specialty to train residents in circumcision. General pediatricians, however, consider it a desirable procedure to learn.^3^ Other providers are also known to perform the procedure, including advanced practice providers, family medicine physicians, obstetricians, surgeons, and urologists.^4,5^ Because the majority of US parents choose circumcision for their male newborns, training in residency is not required, and providers have expressed an interest in learning, a need for circumcision education exists.^6^

Simulation training with deliberate practice is an effective method for acquiring procedural skills.^7^ A variety of simulation-based teaching and learning methods on neonatal circumcisions have been reported.^8^ Strategies include independent learning from web-based content followed by review with a mentor,^9,10^ and use of models ranging from organic material (such as cocktail wieners sheathed in the finger cut-out of a medical glove^11^) to low- and high-fidelity simulators (such as a pacifier and clipboard model to a model simulating a moving and crying infant).^12,13^ For procedures not required during residency, yet commonly performed, exposure via simulation can be beneficial.^3,13^ Personal and professional pursuits beyond trainee years vary considerably. As the procedural skills needed for one's chosen career emerge, mastery-level competency can then grow with deliberate practice.^14,15^ With this in mind, we report our experience carrying out a neonatal circumcision simulation session in a large pediatric residency program.

In our hospital system, circumcisions are taught during the newborn medicine rotation. With the recent addition of a 3D printer to our simulation center and interest in enhancing neonatal circumcision training, a session was developed using 3D-printed models. Tasked to work within a limited time frame and capitalizing on the small-group format of orientation day, we designed a simulation primer to expose interns to the tools and steps involved in performing a circumcision via Gomco clamp. We used Sawyer's pedagogical framework of learn, see, practice, prove, do, maintain for procedural skill training in medicine and addressed the first three elements in an introductory do-along session where learners followed alongside the instructor through a checklist of steps.^16^ The aim was to support interns through the first phase of psychomotor learning and to facilitate self-identification of steps to consider for deliberate practice.^15^ Our educational objectives were for interns to demonstrate the steps, self-identify which steps were more technically challenging to perform, and increase their confidence with the procedure.

## Methods

### Development

Our simulation session was carried out at a large, freestanding, tertiary care children's hospital. Each year, the incoming pediatric residency class includes over 50 interns from across the country. This includes trainees who are in the categorical pediatrics program, a combined program with internal medicine, neurology, or medical genetics, in addition to a primary care track cohort. All trainees participate in the newborn medicine rotation during the intern year. Incorporated into orientation, prior to the first day of residency, is a procedure day in the hospital simulation center, during which small groups of 5–6 interns rotate through different stations, each 30 minutes long. Newly introduced in 2023 was our circumcision simulation station. No prerequisite knowledge specific to circumcisions or procedures was expected of participants. Facilitators included two pediatric hospitalists and one senior resident (all three authors of this report). Our team of facilitators had collectively over 15 years of experience performing over 2,000 circumcisions using both the Gomco clamp and Plastibell device. In addition, the sessions included a near-peer facilitator (Jennifer Springer) who brought a perspective more attuned to our beginner participants.

### Equipment/Environment

Tables were set up in the available space, creating stations for each participant and facilitator. There was a total of eight stations, two for facilitators, six for participants. Participants were positioned on either side of the facilitator to easily allow everyone to go through the steps together. Each station had a 3D-printed model taped to the table. Printing instructions for the model can be found in Appendix A.^17^ A water balloon with a small opening cut at the tip (to simulate the urethral meatus) was placed over the 3D model to represent the foreskin. Gomco circumcision kits, which included two straight hemostats, one curved hemostat, one pair of scissors, one scalpel, and one Gomco clamp sized 1.1 cm, were available at each station (Appendix B). A checklist of the procedure steps was taped next to each 3D model (Appendix C). A third facilitator (Jennifer Springer) helped participants troubleshoot during the 30-minute session. The session was repeated to accommodate all interns. With the exception of a newly positioned balloon foreskin, the same materials were reused each time.

### Personnel

For an optimal experience, two facilitators (Rakhi Gupta Basuray and Sofia Davila) knowledgeable in neonatal circumcisions led the small group through discussion and demonstration of a circumcision. A third facilitator (Jennifer Springer), comfortable with the steps of the procedure, was valuable in helping participants on an individualized basis. The curriculum developers also served as the facilitators.

### Implementation

Prior to the session, participants were emailed instructions to review a video demonstrating the steps of the circumcision simulation (Appendix D). The 6-minute video followed a 15-step checklist developed by the session facilitators (Rakhi Gupta Basuray and Sofia Davila) with input from the newborn medicine rotation attendings. The in-person simulation session agenda began with introductions and review of the simulation session objectives (Appendix E). The session progressed through handling of the instruments and ended with a step-by-step demonstration of the procedure checklist steps as participants followed along. A dedicated debrief period was reserved prior to concluding. A follow-up email with a brief review article on circumcisions was additionally provided.^1^

### Debriefing

Throughout the demonstration and do-along session, participants were able to ask clarifying questions and obtain feedback with each step. Once the simulation concluded, participants shared aspects that were challenging, aspects that were beneficial, and aspects recommended for change.

### Assessment

A QR code was provided on the procedure checklist at each station inviting participants to complete a post-simulation survey (Appendix F). The survey included 16 questions covering past experiences, feedback on specific aspects of the simulation session, rating of one's confidence, and overall scoring of the session. Response options varied from *yes*/*no* to 5-point Likert-scales for their respective questions (ratings 1–5, with low ratings representing descriptors such as *less helpful*, *very confusing*, *unaware of the steps*, *very low*, *unnecessary*, and *poor*, and high ratings representing descriptors such as *very helpful*, *very clear*, *very comfortable*, *very high*, and *excellent*). An opportunity to provide free-text comments was also offered.

The survey was designed to address and measure the educational objectives of the session. The optional survey was approved by the institutional review board (IRB) of Nationwide Children's Hospital and qualified as exempt due to minimal risk (IRB Study ID 00003166). The purpose of the survey was to measure the effectiveness of the simulation session in achieving the stated objectives. The survey took fewer than 5 minutes to complete. Reminder emails to voluntarily and anonymously complete the survey were sent to participants twice after the end of the simulation day.

## Results

The circumcision simulation session using Gomco clamp was available to 54 incoming intern residents. The final count of participants was 53 due to an unexpected conflict arising for one intern. The optional survey offered following the simulation was started by 42 participants and completed in entirety by 38, yielding a survey completion rate of 72% (38/53).

Overall, the simulation session was rated an average 4.9 (range 4–5) on 5-point Likert scale. When participants were asked how helpful the session was to training, the average rating was 4.8 (range 3–5). Survey results identified two participants (5%) with previous experience performing simulated circumcisions, albeit more than 1 year prior, whereas seven participants (17%) had previously completed a real-life circumcision on a newborn.

The helpfulness of the presimulation instructional video for learning the checklist steps of the procedure was rated an average 3.9 (range 1–5). The clarity of the checklist of procedure steps was rated an average 4.7 (range 1–5). Prior to the simulation session, participants rated their comfort level with the steps of the procedure as an average 1.5 (range 1–4). After the simulation session, confidence levels with performing a Gomco clamp circumcision on a patient increased to an average rating of 3.4 (range 2–5). Survey respondents, when asked to answer questions with *yes* or *no*, were unanimous that the session improved their confidence in being able to successfully perform a circumcision, that the simulation session was helpful to experience prior to performing a circumcision on a newborn, and that the session allowed identification of steps for targeted improvement.

The procedure steps on the checklist and those identified by survey respondents as most challenging to perform are summarized in the Table. Excising the foreskin was selected as most challenging by 39% of respondents, followed by a three-way tie between the steps of *Clamping the edges of the dorsal slit together to secure the bell*, *Delivering the foreskin through the underside of the baseplate*, and *Grasping the foreskin free edges on top of the baseplate*, each being selected as most challenging by 13% of survey respondents.

**Table.**
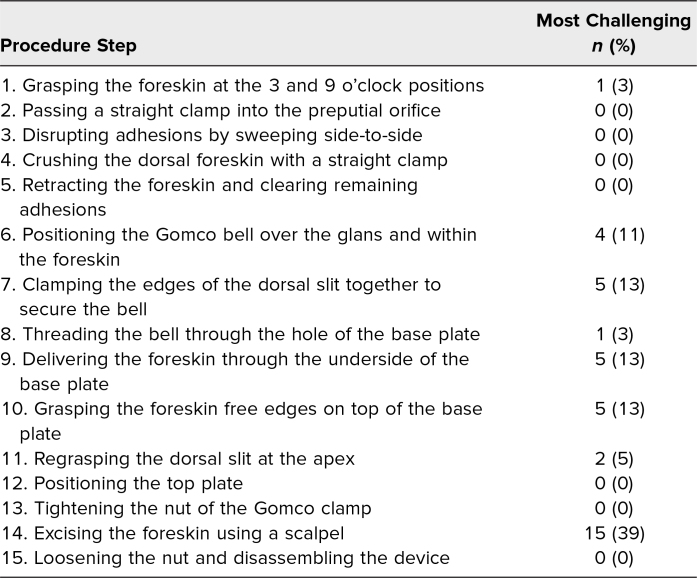
Circumcision Steps and Those Identified as Most Challenging by Participants (*N* = 38)

Free-text responses on what participants liked about the session included the following: “An opportunity to practice the steps and tools;” “Very clear directions and steps;” “Slow and encouraging (instruction/facilitators);” and “Completing steps together with instructors.” Dislikes mentioned on free-text responses included the following: not liking the presimulation video; wanting more time to practice after the simulation; difficulty working with the “flimsy” material of the balloon; and not being able to adjust the height of the procedure table.

## Discussion

A neonatal circumcision simulation session within a large pediatric residency program was successfully and efficiently completed. An overarching objective was to expose participants to a high-demand procedure without the added pressure of attaining competency. The high overall rating of the session reflects that participants were receptive to this. Moreover, the unanimous affirmation that the session was helpful prior to performing circumcision on a newborn, that the session allowed identification of areas for focused improvement, and that the session improved confidence aligns with what has been previously reported on the value of simulation training on procedural skills.

Our survey results provide further insight into the specific steps of a Gomco clamp circumcision that participants found most challenging. This information was helpful to incorporate into the scripting of future instruction; for example, facilitators noted that “these next steps are the most difficult; do not worry about needing to take more time here.” Also important to note is the adaptability and high-value nature of the session. Small groups of any professional background could complete the training in 30 minutes, with repeat sessions as needed for larger groups, and could be conducted at a minimal cost due to reusable tools and inexpensive supplies.

The idea of a circumcision simulation session stemmed from one individual and grew as a result of stakeholder investment in medical education. The concept evolved to meet preexisting needs. We decided to use the Gomco clamp based on current practice on the newborn medicine rotation. This helped in gaining buy-in from respective attendings. The agenda of the session was adapted to fit the constraints of the intern orientation day schedule. The 30-minute period became a welcomed challenge into which the session was fit. The model was decided after a coincidental discovery of the capabilities of the simulation center 3D printer. In the absence of a 3D printer, a pacifier could be an alternative model. The addition of a third facilitator (Jennifer Springer), a current resident, was instrumental in providing an alternative perspective. This also helped ensure a smooth flow to the session by having a dedicated instructor float between individuals to support as needed. One lesson learned from participant feedback was that the supplemental prerecorded video of the simulation was not valued as highly as the hands-on experience. We maintain, however, that having the option to access such a resource can be helpful for focused, independent practice. To support this, a station was set up in the nursery so that trainees could electively practice their skills during their newborn medicine rotation. Lastly, comments from participants revealed that the flimsy material of the balloon may have influenced the identification of the most challenging step of the procedure. Since the initial development of the session, more resilient water balloons have been secured.

While demonstrating mastery of procedural skills is best for patient safety, defining competency and creating universal standards suitable for all is complex.^18^ Considering that circumcisions are not a procedure required by the ACGME, but of interest to general pediatricians, our simulation session supports exposing undifferentiated trainees to this procedure. The additional setup of the simulation station in a space for learners to independently practice while on their newborn medicine rotation allowed for deliberate practice, and allowed individuals to follow Sawyer's pedagogical framework for procedural skill training by *proving* their readiness to *do*.^16^ While our simulation session improved confidence of beginner participants, a healthy dose of which is necessary to be successful in clinical practice, it is important to highlight that competency requires repetition beyond an introductory simulation session.^19^ A recently published report additionally details the importance of hands-on circumcision training and structured learning, elements that this simulation session provides.^6^

It is important to acknowledge a few limitations of the session. First, it was not designed to be a comprehensive course on neonatal circumcision as it lacks review of essentials, for example, contraindications, administering anesthesia, and managing complications. Due to the time constraints of the session, it was felt that such a curriculum would not be feasible, and it was further acknowledged that these elements were reviewed during the clinical rotation. Replicating adhesions by simulation was another absent element, and the difficulty in accommodating this element is likely the reason why other circumcision simulation models also do not include it. Additionally, alternative methods of neonatal circumcision, such as the Mogen clamp and Plastibell device, may be practiced by providers within and outside the field of pediatrics. This session, however, could be adapted to be completed by providers of any specialty, with an internally crafted checklist of procedure steps, tools, and materials (i.e., a pacifier instead of a 3D-printed model), while maintaining the same framework described above. Lastly, for half the participants, the simulation session was disconnected from actual practice by more than 6 months. Anecdotally, these residents favorably recalled the experience and appreciated the opportunity to continue practicing during the rotation on the reproduced simulation station available to them.

Looking ahead, following up with participants after in vivo performance will help improve and validate aspects of the simulation session. As ACGME requirements continue to evolve, simulation may play a greater role in providing procedural exposure as senior residents begin to narrow their professional pursuits, fellowships expand, and privileges of advanced practice providers expand.

## Appendices


3D Printing Instructions.stlSupply Checklist.docxProcedure Steps.docxCircumcision Video.mp4Agenda and Facilitator Guide.docxSurvey.docx
*All appendices are peer reviewed as integral parts of the Original Publication.*
