# Supplementary material for: Neonatal Circumcision Simulation: A Resource for Beginners
Source: MedEdPORTAL. 2025 Jun 3;21:11531. doi: 10.15766/mep_2374-8265.11531 (PMC12130306; doi:10.15766/mep_2374-8265.11531)
Supplement: Supplementary file 1 — 3D Printing Instructions.stlSupply Checklist.docxProcedure Steps.docxCircumcision Video.mp4Agenda and Facilitator Guide.docxSurvey.docx [file mep_2374-8265.11531-s001.zip › B. Supply Checklist.docx]

**Circumcision Simulation**

**Supply Checklist for each Station**

One station per participant and demonstrating facilitator

- Procedure Steps, printed and taped to the table
- 3D Model, taped to the table
-
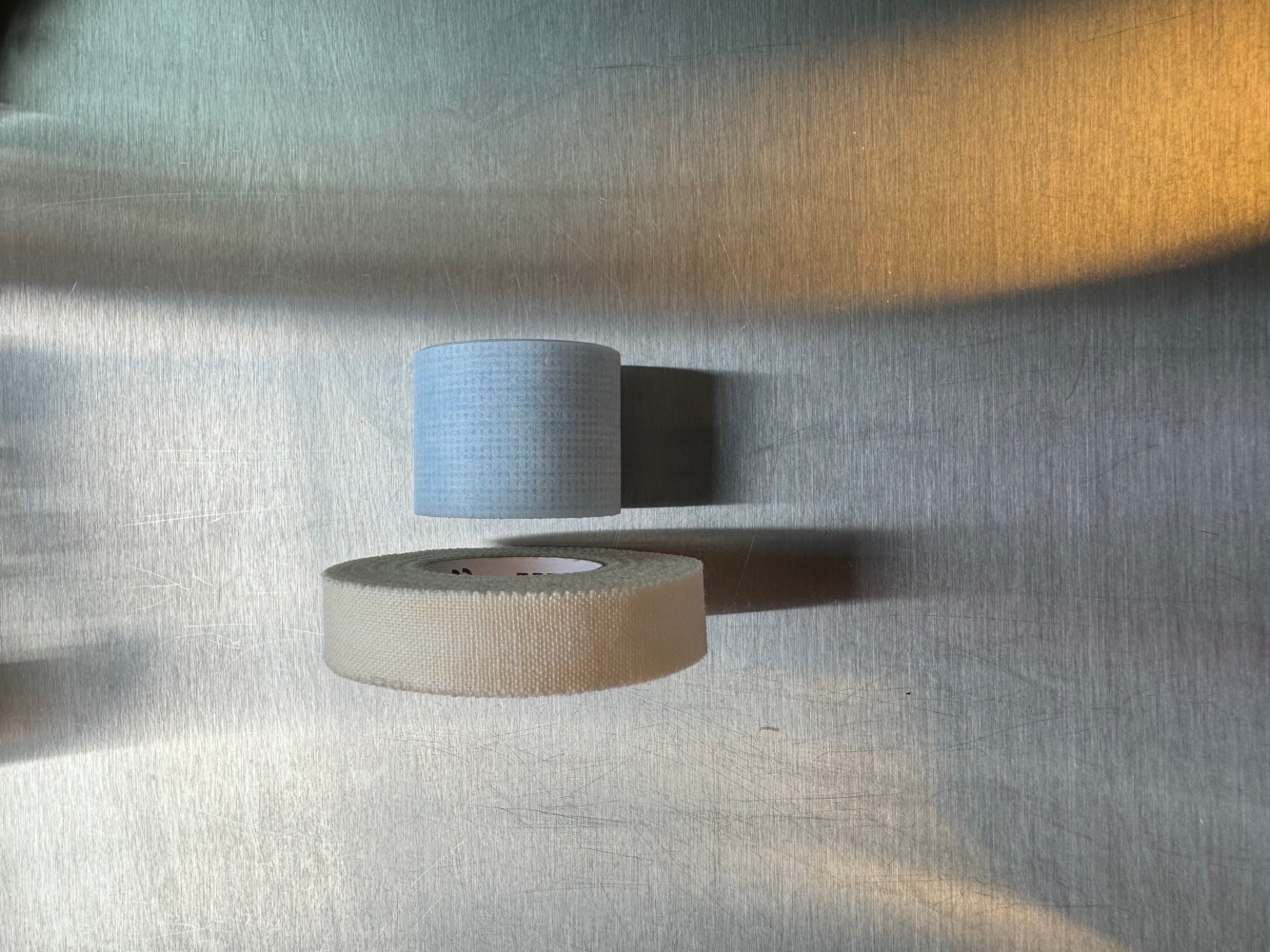
Tape, suitable options
- Water balloon with cut tip, $\geq$2 per participant, we recommend using textured water balloons which are less flimsy

- Gomco clamp, size 1.1cm
- Hemostats, curved x2 (if available)
- Hemostats, straight x2
- Scalpel
- Scissors
- Facilitators: ratio ~ 2 facilitators for ~6 participants (one to demonstrate the procedure and another to walk around during the procedure steps to assist participants)
