# Supplementary material for: Neonatal Circumcision Simulation: A Resource for Beginners
Source: MedEdPORTAL. 2025 Jun 3;21:11531. doi: 10.15766/mep_2374-8265.11531 (PMC12130306; doi:10.15766/mep_2374-8265.11531)
Supplement: Supplementary file 1 — 3D Printing Instructions.stlSupply Checklist.docxProcedure Steps.docxCircumcision Video.mp4Agenda and Facilitator Guide.docxSurvey.docx [file mep_2374-8265.11531-s001.zip › C. Procedure Steps.docx]

**Gomco Circumcision – Simulation Steps**

1. Grasp the foreskin at the 3 and 9 o’clock positions
2. Pass a straight clamp into the preputial orifice
3. Disrupt adhesions by gently sweeping side-to-side
4. Crush the dorsal foreskin with a straight clamp
5. Complete the dorsal slit using scissors
6. Retract the foreskin and clear remaining adhesions
7. Position the bell over the glans and within the foreskin
8. Clamp the edges of the dorsal slit together to secure the bell
9. Thread the bell through the baseplate and deliver the foreskin
10. Grasp the foreskin free edges on top of the base plate
11. Re-grasp the dorsal slit at the apex
12. Position the top plate
13. Tighten the nut of the Gomco clamp
14. Excise the foreskin using a scalpel
15. Loosen the nut and disassemble the device
