# Supplementary material for: Neonatal Circumcision Simulation: A Resource for Beginners
Source: MedEdPORTAL. 2025 Jun 3;21:11531. doi: 10.15766/mep_2374-8265.11531 (PMC12130306; doi:10.15766/mep_2374-8265.11531)
Supplement: Supplementary file 1 — 3D Printing Instructions.stlSupply Checklist.docxProcedure Steps.docxCircumcision Video.mp4Agenda and Facilitator Guide.docxSurvey.docx [file mep_2374-8265.11531-s001.zip › E. Agenda and Facilitator Guide.docx]

Session Objectives

By the end of this activity, learners will be able to:

1. Demonstrate steps of neonatal circumcision via Gomco
2. Self-identify steps of neonatal circumcision more technically challenging to perform
3. Increase confidence in performing neonatal circumcision

**Agenda**

30 Minute Session

- Introductions of facilitators and participants (5 minutes)
  - Discussion of circumcision video, previous simulation experience, and if any prior experience with actual neonatal circumcisions.
  - Review of above objectives and that the focus here is on steps of the procedure
- Introduction to each participants’ station (5 minutes)
  - Explanation of model and balloon foreskin
  - Clarification on right or left-handedness
  - Review of circumcision instruments and handling of each tool
  - Safety instructions on “sharps”
- Initiation through completion of all steps of the procedure checklist (15 minutes)
- Debrief (5 minutes)
  - What went well
  - What was difficult
- **Introductions of facilitators and participants (5 minutes)**
  - **Discussion of circumcision video, previous simulation experience, and if any prior experience with actual neonatal circumcisions.**
    - Ask if anyone was able to review the video in advance. It was not unusual that someone was unable, due to access barriers, time, or overall awareness.
    - Provide reassurance to those who did, or did not view in advance, that it is encouraged to view the video after the session and again as they approach a rotation where they may complete circumcisions, and even after having done the procedure. Different pointers may be picked up at different stages of familiarity with the procedure.
    - Allow participants to share their experience with circumcisions and simulation, to better understand where everyone may be coming from. We’ll be going over just one approach but there are other ways to perform a circumcision with similar outcomes.
  - **Review of above objectives and that the focus here is on steps of the procedure**
    - Emphasize that today’s session was designed to be compact and to allow participants to zero in on one specific aspect of circumcisions, becoming acquainted with the tools and sequence of the steps of the procedure.
    - An excellent review on “Newborn Circumcision Techniques,” including additional elements such as anatomy, anesthesia, various clamps, post-procedure care, complications and contraindications is freely available online by the American Academy of Family Physicians. (Omole F, Smith W, Carter-Wicker K. Newborn Circumcision Techniques. *Am Fam Physician*. 2020;101(11):680-685.)
- **Introduction to each participants’ station (5 minutes)**
  - Explanation of model and balloon foreskin
    - A) 3D Model
    - B) 3D Model taped to the table
    - C) Balloon to replicate foreskin
    - D) Balloon on 3D Model taped to the table


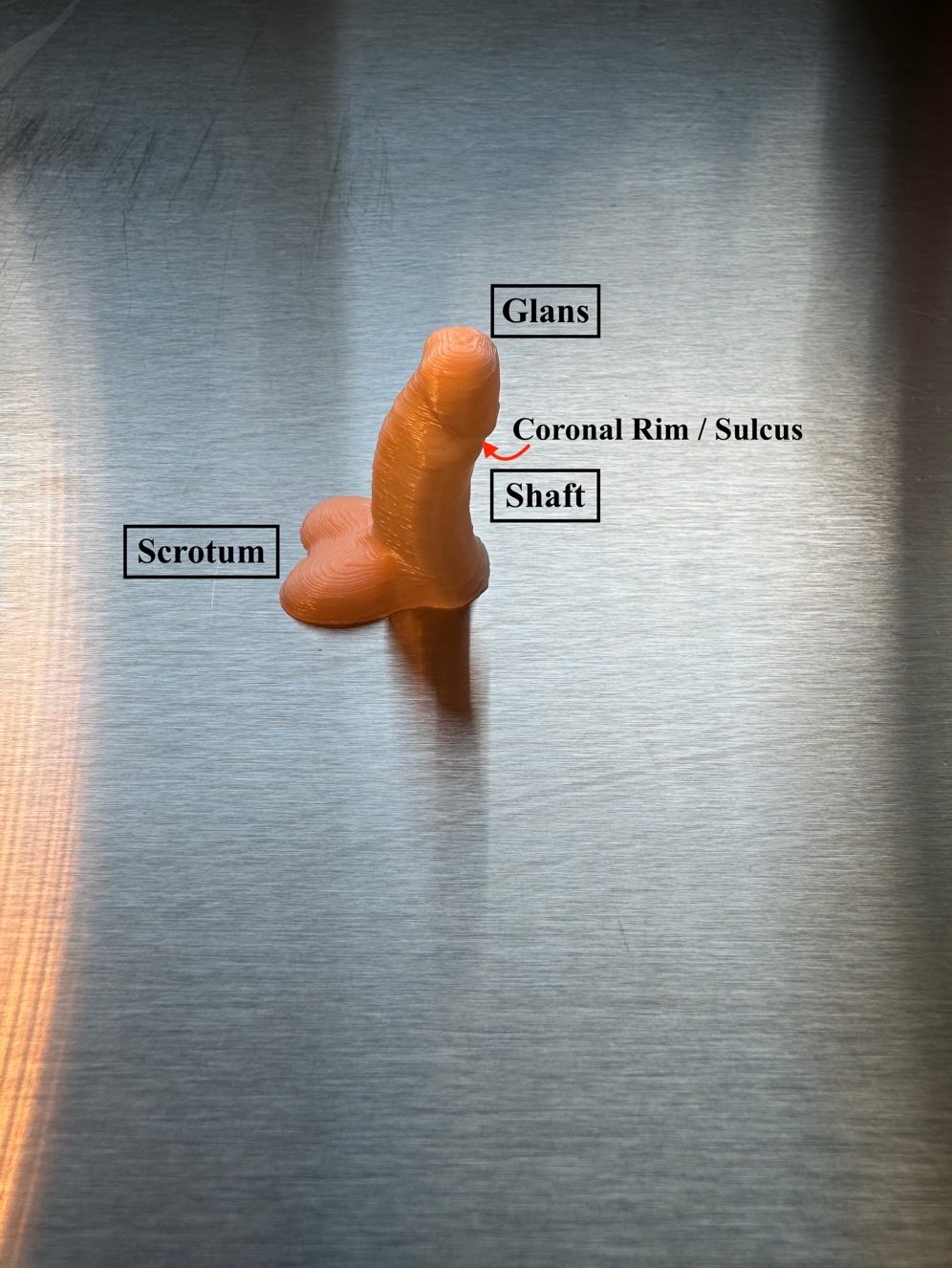

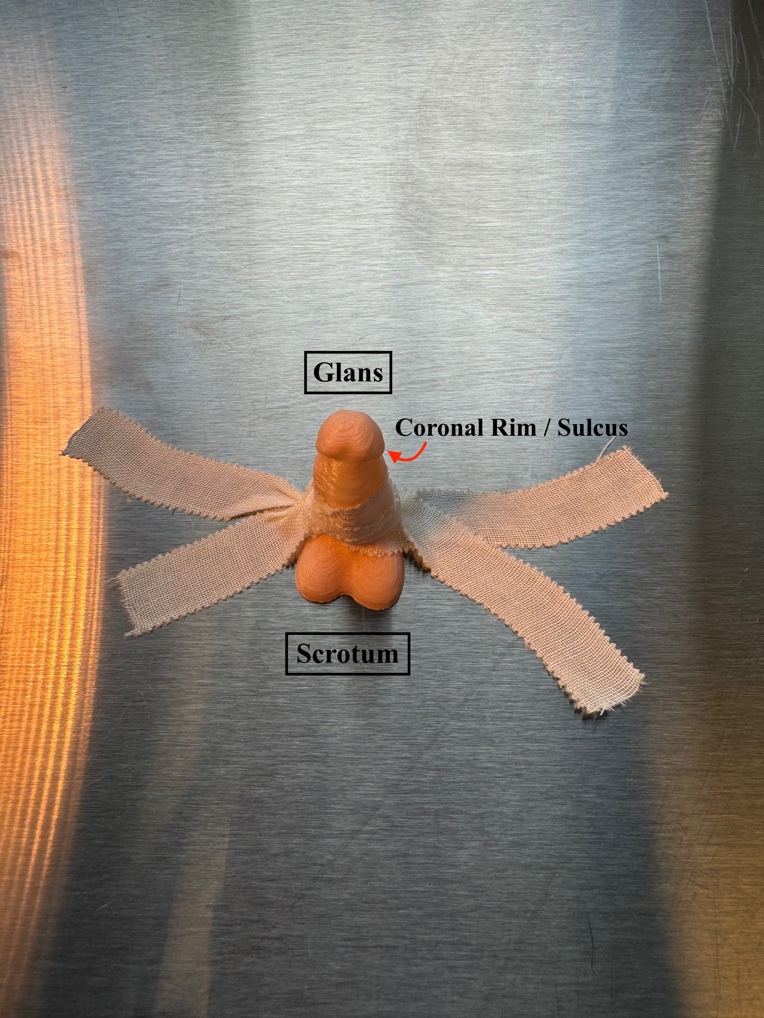

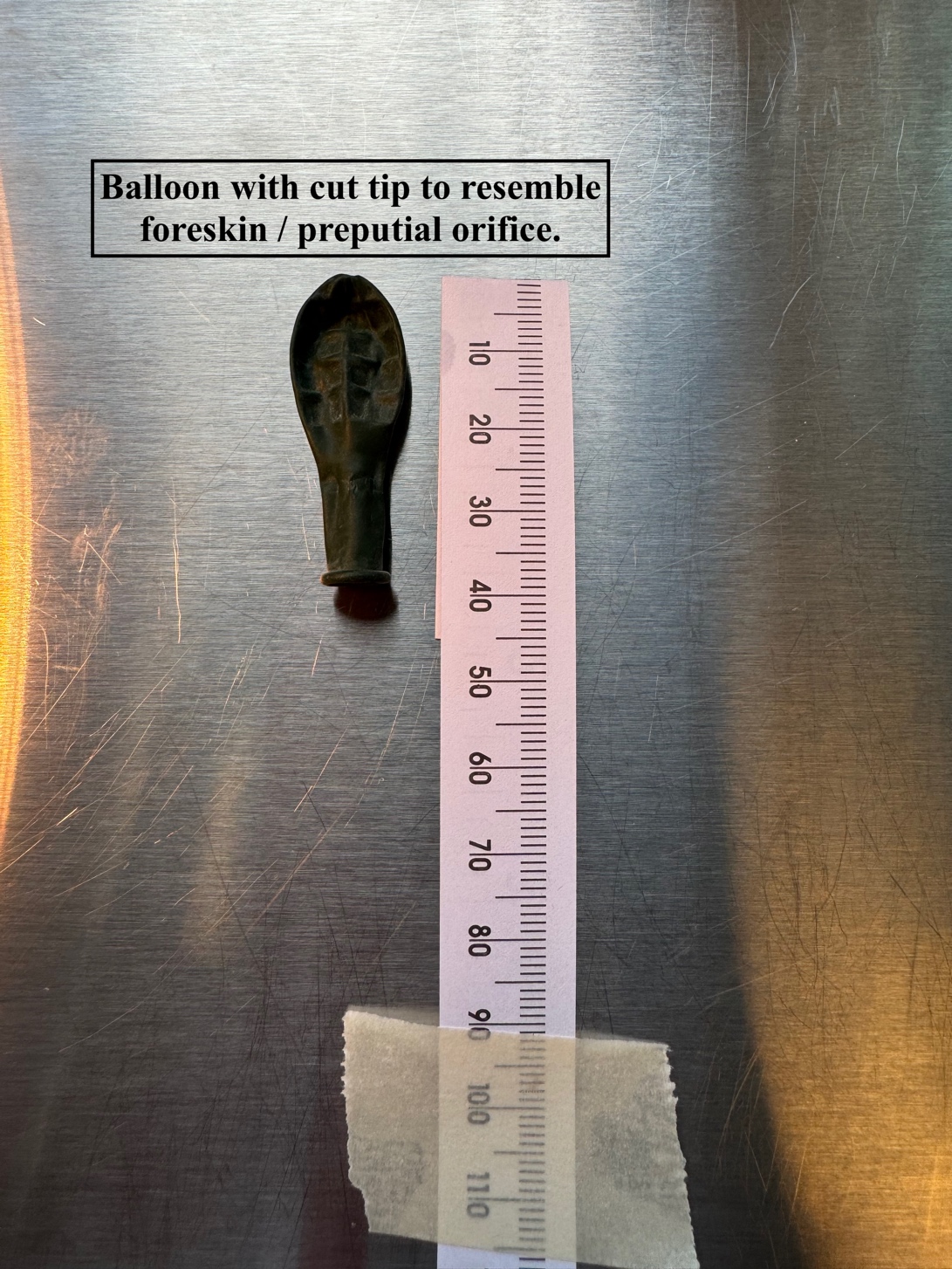

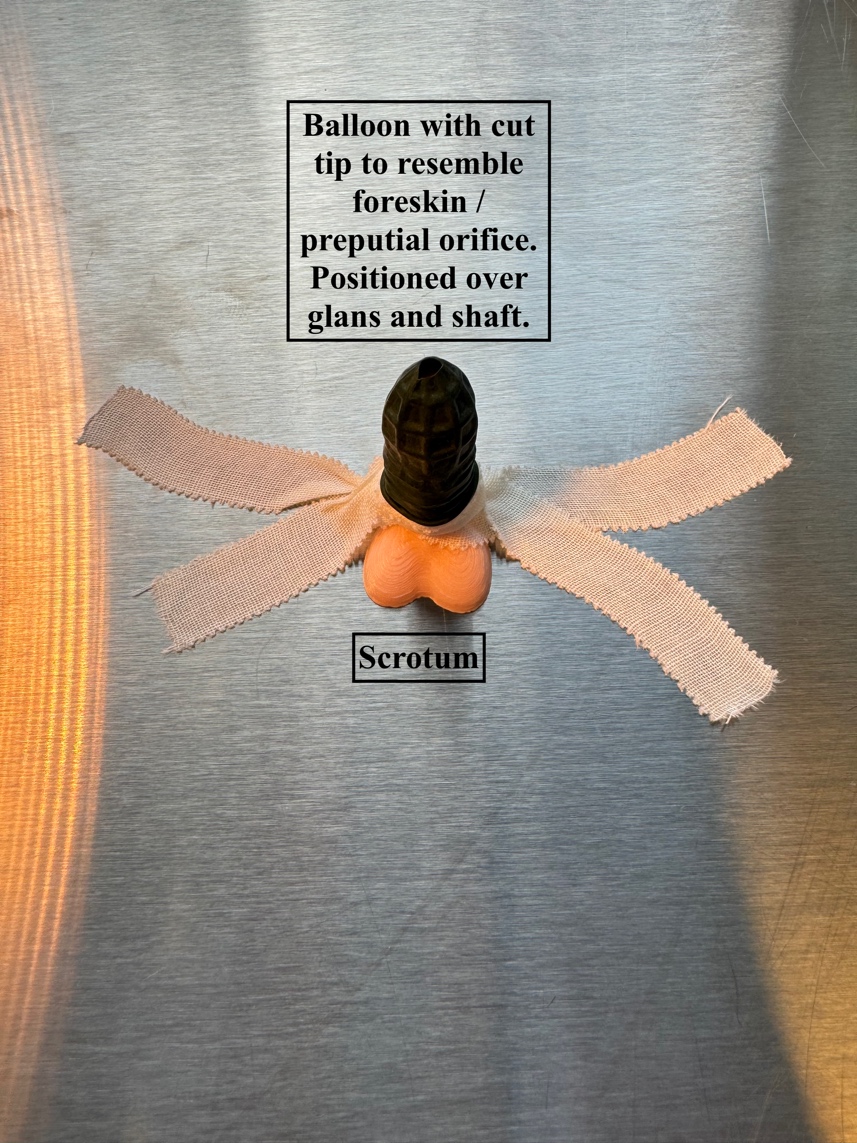


B)

C)

D)

A)

- - **Clarification on right or left-handedness**
    - Individuals with a dominant hand that is opposite that of the demonstrating facilitator may be best positioned opposite to the facilitator, so that they can mirror the steps.
    - When someone is present with a dominant hand that is opposite that of the facilitator, concerted efforts to using “dominant hand” instead of “right” or “left” can help when proceeding through the steps of the procedure.
  - **Review of circumcision instruments and handling of each tool**
    - A) Gomco Clamp Assembled
      - Review terminology below
      - Allow participants to disassemble and reassemble the clamp to become familiar with how it comes together
    - B) Complete set of tools with Gomco disassembled
      - Review terminology below
      - Allow participants to practice opening and closing the hemostats in their dominant and non-dominant hands
      - Allow participants to practice exposing the blade of the scalpel as well as re-capping. Instruct participants to hold the scalpel like a pencil for optimal control.


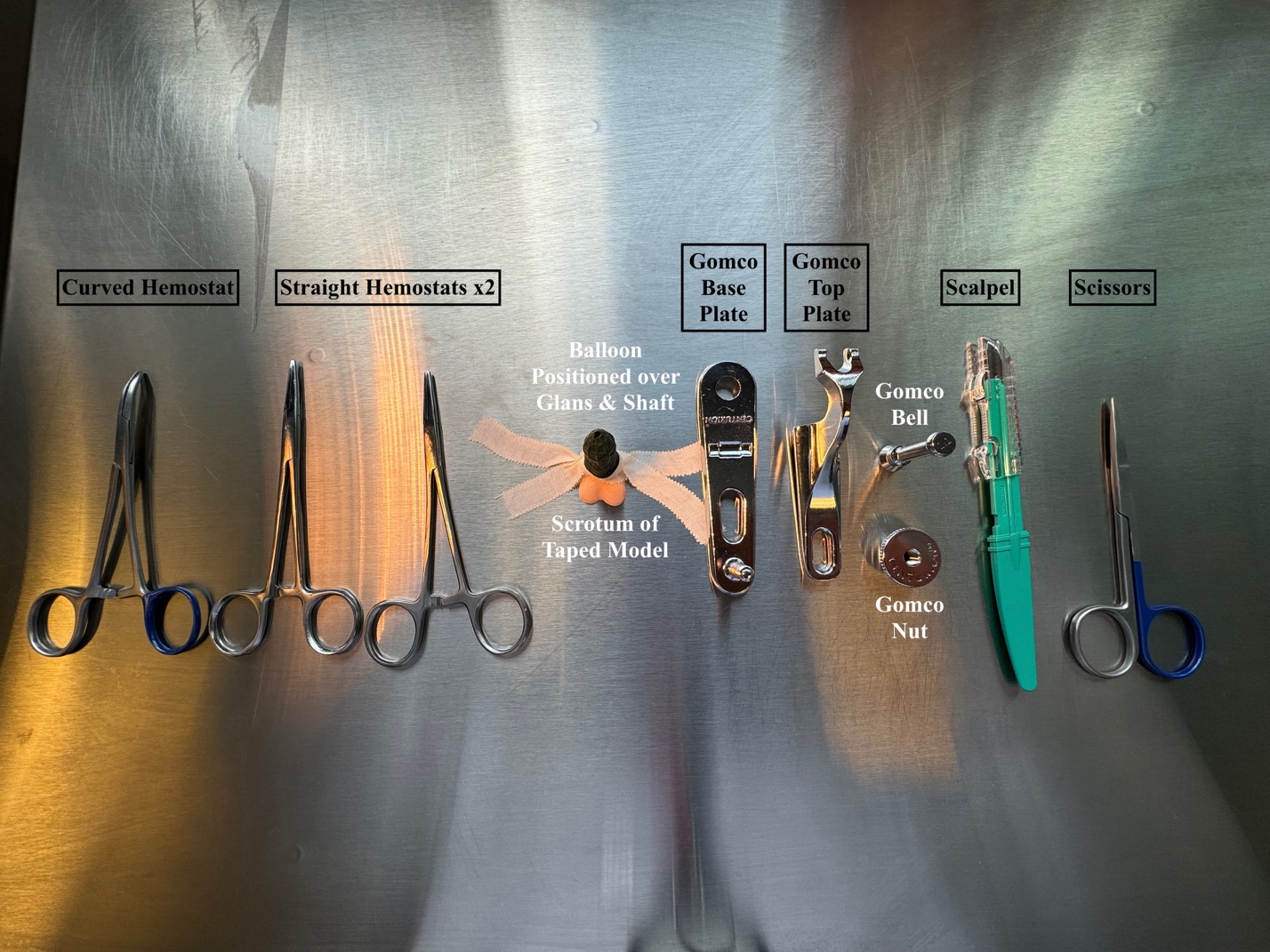


B)


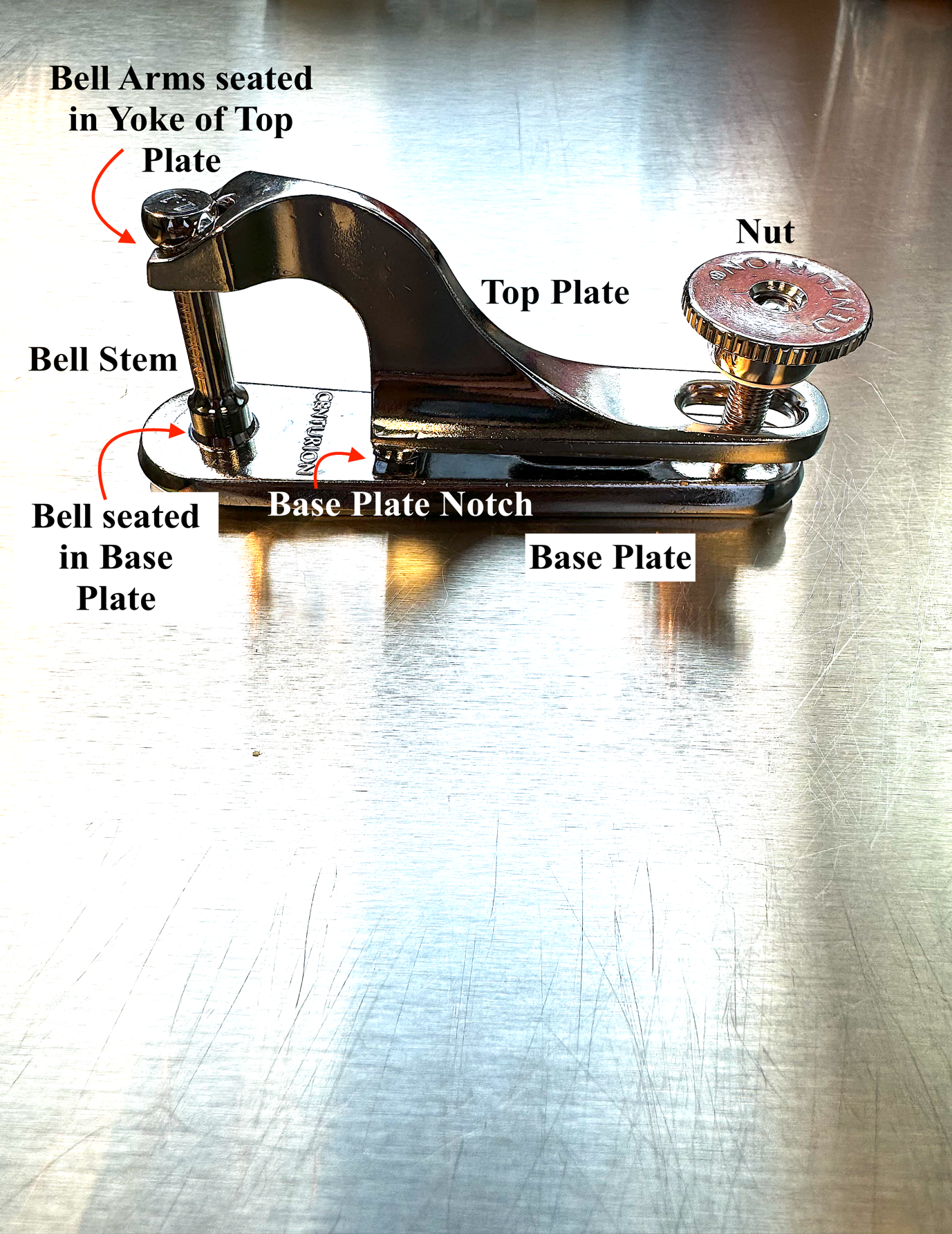


A)

- - **Safety instructions on “sharps”**
    - It is ideal to immediately dispose of syringes, scissors and scalpels in a “sharps” bin once done with the tool during a procedure.
    - Some tools have a safe way to re-cap, those that do not should not be manually re-capped to avoid risk of harming yourself
    - For our purposes, in needing to re-use tools, please work cautiously, keeping in mind the sharps that remain in your field during the procedure.
    - The facilitators can carefully re-set the tools for the next group at the completion of the session.
- **Initiation through completion of all steps of the procedure checklist (15 minutes)**
  - Additional commentary has been added in below for each step of the procedure, and is supported with visuals provided in the video.

1. Grasp the foreskin at the 3 and 9 o’clock positions
   1. Use one curved hemostat and one straight clamp, it is not necessary to grasp the foreskin directly at the preputial orifice
   2. Rest both hemostats in your non-dominant hand, so the tips approximate one another and make the point of an upside-down “V”
   3. Secure the hemostats in place by positioning your non-dominant thumb on top of the hemostats.
2. Pass a straight clamp into the preputial orifice
   1. Provide gentle traction on the foreskin with hemostats in your non-dominant hand
   2. Introduce the third straight clamp, held in your dominant hand, so the tip enters the preputial orifice at the 12 o’clock position, between the other 2 hemostats
   3. Visibly slide the straight clamp along the interior aspect of the foreskin to ensure the clamp does not enter the meatus of the glans
   4. Advance the straight clamp down to the base of the glans
3. Disrupt adhesions by gently sweeping side-to-side
   1. Cautiously open the jaws of the clamp and withdraw the straight clamp entirely
   2. Once your straight clamp has fully exited the space and is entirely visible to your eye, close the straight clamp
   3. Repeat by re-entering the preputial orifice with the tip aiming at 3 o’clock and 9 o’clock, gently open the jaws of the clamp and exit
   4. Avoid the 6 o’clock, ventral aspect of the glans where the frenulum can easily bleed with disruption
4. Crush the dorsal foreskin with a straight clamp
   1. Maintain mild traction with holding the 2 hemostats in your non-dominant hand
   2. With your dominant hand, open the 3^rd^ straight clamp and advance one jaw into the preputial orifice at the 12 o’clock position
   3. Visibly slide the one jaw along the interior aspect of the foreskin ~0.5”
   4. Close the straight clamp and crush the foreskin
5. Complete the dorsal slit using scissors
   1. Remove the straight clamp used to crush the foreskin
   2. Maintain mild traction with holding the 2 hemostats in your non-dominant hand
   3. With your dominant hand, grasp the scissors and cut the foreskin along the imprint made by the clamp. Do not exceed beyond the imprint. The imprint will not be as visible on the balloon foreskin simulation.
6. Retract the foreskin and clear remaining adhesions. (In this simulation, after retracting the foreskin there are no simulated adhesions to clear.)
   1. Remove the original 2 hemostats in place
   2. Using gauze, retract the foreskin and fully expose the glans to visualize the coronal sulcus
   3. Re-grasp the foreskin at the 3 and 9 o’clock positions using two straight clamps
7. Position the Gomco bell over the glans and within the foreskin
   1. With your dominant hand, position the Gomco bell over the glans
   2. Keeping the bell vertically in position, envelop the bell by returning the foreskin held by the 2 hemostats to their anatomical position
8. Clamp the edges of the dorsal slit together to secure the bell
   1. Allow the 2 straight clamps to rest in your non-dominant hand with mild traction applied
   2. Stabilize the bell vertically with your non-dominant thumb
   3. With the curved hemostat in your dominant hand, clamp the edges of the dorsal slit together at the stem of the Gomco bell
   4. Remove the 2 straight clamps
9. Thread the bell through the baseplate and deliver the foreskin
   1. Introduce the base plate and thread the arms of the bell through the underside of the hole
   2. Deliver the foreskin held together by the curved hemostat through as well
10. Grasp the foreskin free edges on top of the base plate
    1. Maintain hold of the curved hemostat on the underside of the base plate in your dominant hand
    2. With a straight clamp in your non-dominant hand, grasp the free edges of the foreskin on top of the base plate
11. Re-grasp the dorsal slit at the apex
    1. Once the straight clamp has grasped the free edges on top, remove the curved hemostat from below the base plate
    2. Keeping the straight clamp in your non-dominant hand, apply gentle traction to visualize the apex of the dorsal slit
    3. With the 2^nd^ straight clamp in your dominant hand, re-grasp the dorsal slit at the apex, on the top side of the baseplate
    4. Remove the previously positioned straight clamp
12. Position the top plate
    1. With your non-dominant hand, support the bell into an upright position from below the base plate
    2. With your dominant hand, position the top plate by angling the yoke downwards to catch the arms of the bell
13. Tighten the nut of the Gomco clamp
    1. Allow the top plate to rest in the notch of the base plate
    2. If separate, position the washer, then tighten the nut firmly
14. Excise the foreskin using a scalpel
    1. With your non-dominant hand, prevent the Gomco from toppling
    2. With the scalpel in your dominant hand, excise the foreskin around the bell
15. Loosen the nut and disassemble the device

- Debrief (5 minutes)
  - What went well
    - Congratulate everyone on completing a simulated circumcision! Some aspects of the simulation may be easier than real life and some aspects may be more difficult.
    - Simulation of adhesions and the layers of the foreskin (epithelial and mucosal), which can separate in real life, were unable to be simulated and can make an *in vivo* circumcision more difficult.
  - What was difficult
    - The flimsy and thin nature of the water balloons can make the “foreskin” difficult to grasp with the hemostats or allow for the “foreskin” to tear easily.
