# Supplementary material for: Neonatal Circumcision Simulation: A Resource for Beginners
Source: MedEdPORTAL. 2025 Jun 3;21:11531. doi: 10.15766/mep_2374-8265.11531 (PMC12130306; doi:10.15766/mep_2374-8265.11531)
Supplement: Supplementary file 1 — 3D Printing Instructions.stlSupply Checklist.docxProcedure Steps.docxCircumcision Video.mp4Agenda and Facilitator Guide.docxSurvey.docx [file mep_2374-8265.11531-s001.zip › F. Survey.docx]

**Gomco Circumcision Simulation Survey**

1. Have you completed previous simulation training in circumcision procedures? Yes / No
2. How recent was the simulation training completed? 0-6 months ago / >12 months ago / N/A
3. Have you completed a circumcision on a newborn? Yes / No
4. On a scale of 1-5, how helpful was the video prior to the simulation session for learning the steps of the Gomco Circumcision Procedure Checklist?
   1. 1 – Very unhelpful!
   2. 2 – Not helpful
   3. 3 – Neutral
   4. 4 – Helpful
   5. 5 – Very helpful!
   6. N/A
5. On a scale of 1-5 how clear were the steps of the Gomco Circumcision Procedure checklist?
   1. 1 – Very Unclear!
   2. 2 – Not clear
   3. 3 – Neutral
   4. 4 – Clear
   5. 5 – Very Clear!
6. The most challenging aspect of the procedure was: Steps 1 – 15 provided as options
7. Prior to the circumcision simulation, I would rate my comfort level in the steps of the Gomco Circumcision Procedure as:
8. 1 – Very Uncomfortable!
9. 2 – Not comfortable
10. 3 – Neutral
11. 4 – Comfortable
12. 5 – Very Comfortable!
13. After completing the circumcision simulation, I would rate my confidence with performing a Gomco Circumcision on a patient to be:
    1. 1 – Very Low!
    2. 2 – Low
    3. 3 – Neutral
    4. 4 – High
    5. 5 – Very High!
14. The simulation session improved my confidence in being able to successfully perform a circumcision: Yes / No
15. The session allowed me to identify steps of the procedure in which I need to improve: Yes / No
16. The simulation was helpful to experience prior to performing a circumcision on a newborn: Yes / No
17. On a scale of 1-5 how helpful was the Gomco Simulation for your training?
    1. 1 – Very Unhelpful!
    2. 2 – Not helpful
    3. 3 – Neutral
    4. 4 – Helpful
    5. 5 – Very helpful!
18. Overall rating of the circumcision simulation session:
    1. 1 – Terrible!
    2. 2 – Poor
    3. 3 – Neutral
    4. 4 – Good
    5. 5 – Excellent!
19. One thing I liked about the session: Free text
20. One thing I disliked about the session: Free text
21. Additional comments: Free text
